# Genome-wide association study of chronic sputum production implicates loci involved in mucus production and infection

**DOI:** 10.1183/13993003.01667-2022

**Published:** 2023-06-15

**Authors:** Richard J. Packer, Nick Shrine, Robert Hall, Carl A. Melbourne, Rebecca Thompson, Alex T. Williams, Megan L. Paynton, Anna L. Guyatt, Richard J. Allen, Paul H. Lee, Catherine John, Archie Campbell, Caroline Hayward, Maaike de Vries, Judith M. Vonk, Jonathan Davitte, Edith Hessel, David Michalovich, Joanna C. Betts, Ian Sayers, Astrid Yeo, Ian P. Hall, Martin D. Tobin, Louise V. Wain

**Affiliations:** 1 Department of Population Health Sciences, University of Leicester, Leicester, UK; 2 Leicester NIHR Biomedical Research Centre, Glenfield Hospital, Leicester, UK; 3 Centre for Respiratory Research, NIHR Nottingham Biomedical Research Centre, School of Medicine, Biodiscovery Institute, University of Nottingham, Nottingham, UK; 4 Centre for Genomic and Experimental Medicine, Institute of Genetics and Cancer, University of Edinburgh, Western General Hospital, Edinburgh, UK; 5 Medical Research Council Human Genetics Unit, Institute of Genetics and Cancer, University of Edinburgh, Edinburgh, UK; 6 University of Groningen, University Medical Center Groningen, Department of Epidemiology and Groningen Research Institute for Asthma and COPD (GRIAC), Groningen, The Netherlands; 7 GSK R&D, Collegeville, PA, USA; 8 GSK R&D, Stevenage, UK

## Abstract

**Background:**

Chronic sputum production impacts on quality of life and is a feature of many respiratory diseases. Identification of the genetic variants associated with chronic sputum production in a disease agnostic sample could improve understanding of its causes and identify new molecular targets for treatment.

**Methods:**

We conducted a genome-wide association study (GWAS) of chronic sputum production in UK Biobank. Signals meeting genome-wide significance (p<5×10^−8^) were investigated in additional independent studies, were fine-mapped and putative causal genes identified by gene expression analysis. GWASs of respiratory traits were interrogated to identify whether the signals were driven by existing respiratory disease among the cases and variants were further investigated for wider pleiotropic effects using phenome-wide association studies (PheWASs).

**Results:**

From a GWAS of 9714 cases and 48 471 controls, we identified six novel genome-wide significant signals for chronic sputum production including signals in the human leukocyte antigen (HLA) locus, chromosome 11 mucin locus (containing *MUC2*, *MUC5AC* and *MUC5B*) and *FUT2* locus. The four common variant associations were supported by independent studies with a combined sample size of up to 2203 cases and 17 627 controls. The mucin locus signal had previously been reported for association with moderate-to-severe asthma. The HLA signal was fine-mapped to an amino acid change of threonine to arginine (frequency 36.8%) in HLA-DRB1 (HLA-DRB1*03:147). The signal near *FUT2* was associated with expression of several genes including *FUT2*, for which the direction of effect was tissue dependent. Our PheWAS identified a wide range of associations including blood cell traits, liver biomarkers, infections, gastrointestinal and thyroid-associated diseases, and respiratory disease.

**Conclusions:**

Novel signals at the *FUT2* and mucin loci suggest that mucin fucosylation may be a driver of chronic sputum production even in the absence of diagnosed respiratory disease and provide genetic support for this pathway as a target for therapeutic intervention.

## Abstract 2

**Genome-wide association study in UK Biobank identifies six novel loci associated with chronic sputum production at genome-wide significance in a disease agnostic population. These include a *FUT2* locus, highlighting a possible target for drug development.** https://bit.ly/3IRVJeT

## Introduction

Increased sputum production impacts on daily activities and quality of life, and is a shared feature of many respiratory diseases. Worldwide, 545 million people have chronic respiratory conditions, with those associated with chronic sputum production including COPD, asthma, bronchiectasis, chronic bronchitis and cystic fibrosis. Chronic respiratory disease is the third leading cause of death worldwide, with 3.91 million deaths in 2017 [1].

The determinants of chronic sputum production in disease are not completely understood [2]. Most studies of excess sputum production have been in subjects with chronic bronchitis and COPD where it has been associated with lower lung function [3, 4] and higher risk of both exacerbation and respiratory symptoms [5]. Risk factors for excess sputum production include smoking and occupational and environmental pollutants [4, 6–8]. Currently available drug treatments for those with chronic sputum production do not generally affect the rate of production of sputum, but act as mucolytics and expectorants [9–11].

Genome-wide association studies (GWASs) have highlighted pathways underlying a range of respiratory traits and diseases, and highlighted potentially relevant drug targets [12, 13]. Previous GWASs of sputum production [14–17] have not identified any genome-wide significant findings.

We hypothesised that identifying genetic variants that are associated with chronic sputum production in a large general population sample could improve understanding of its causes and identify new molecular targets for treatment. To test this hypothesis, we undertook a GWAS of risk of chronic sputum production in 9714 cases and 48 471 controls from UK Biobank, and sought replication of the association signals in five additional independent studies totalling 2203 cases and 17 627 controls. We performed phenome-wide association studies (PheWASs) and interrogation of gene expression data to characterise the association signals and determine which genes may be driving these signals.

## Methods

### Study population

Information about chronic sputum production was obtained from the online lifetime occupation survey that was e-mailed to 324 653 UK Biobank participants with existing e-mail addresses between June and September 2015 and achieved a response rate of 38% (31% of all of those contacted provided a full completion of the questionnaire [18]). For this study, we defined cases as those who answered “Yes” to the question “Do you bring up phlegm/sputum/mucus daily?” (UK Biobank data-field 22504; a total of 121 283 participants provided a “Yes” or “No” response). Controls were defined as those who answered “No” to this question. Cases and controls were further restricted to those of genetically determined European ancestry, as previously defined [19], with available smoking data (UK Biobank data-field 20160). Related individuals were removed, with cases preserved over controls when excluding one of a pair (or more) of related individuals (UK Biobank data-field 22021; “related” defined as a KING kinship coefficient ≥0.0884, equivalent to second-degree relatedness or closer). For related pairs within the cases or controls, the individual with the lowest genotype missingness (UK Biobank data-field 22005) was retained. From all available controls, we defined a subset of controls with a similar age (UK Biobank data-field 34) and sex (UK Biobank data-field 31) distribution to the cases at a 1:5 ratio with the cases.

Demographics and respiratory characteristics of the case and controls were derived using the following definitions: doctor-diagnosed asthma (UK Biobank data-field 22127), moderate-to-severe asthma (as previously described [20]), doctor-diagnosed chronic bronchitis (UK Biobank data-field 22129), cough on most days (UK Biobank data-field 22502), smoking status (UK Biobank data-field 20160), COPD Global Initiative for Chronic Obstructive Lung Disease (GOLD) stage 1–4 and stage 2–4 (defined using baseline spirometry as previously described [19, 21]), and bronchiectasis and cystic fibrosis (supplementary tables S1 and S2).

UK Biobank has ethical approval from North West – Haydock Research Ethics Committee (21/NW/0157). Written informed consent was provided by all participants.

### GWAS of chronic sputum production

Genetic data from the v3 March 2018 UK Biobank data release, imputed to the Haplotype Reference Consortium panel r1.1 2016, were used for the GWAS, giving 27 317 434 variants for analysis.

Association testing was performed using logistic regression under an additive genetic model in PLINK 2.0 [22] with age, sex, array version, never/ever-smoking status and the first 10 principal components of ancestry as covariates. Variants were excluded if they had an imputation quality INFO score <0.5 or a minor allele count (MAC) <20. Association signals were considered genome-wide significant at p<5×10^−8^. Independent signals were initially defined using a 1-Mb window (500 kb each side of the sentinel variant) and then using conditional analyses implemented in GCTA-COJO [23]. All variant coordinates are for genome build GRCh37. Region plots were created using LocusZoom [24].

### Replication

We sought replication in five general population cohorts which surveyed participants for chronic sputum production: Generation Scotland [25], EXCEED Study [26], LifeLines 1, LifeLines 2 and Vlagtwedde-Vlaardingen [17]. Further details are provided in the supplementary material.

In addition, the overlap of primary care sputum codes with the chronic sputum production question (UK Biobank data-field 22504) was evaluated to identify whether primary care codes could be used to define an additional independent case–control dataset from those in UK Biobank who did not respond to the online lifetime occupation survey (supplementary material).

### Fine-mapping

We undertook Bayesian fine-mapping for all genome-wide significant signals that were not in the human leukocyte antigen (HLA) region to define 99% credible sets of variants, *i.e.* sets of variants that are 99% probable to contain the true causal variant (assuming that it has been measured).

To fine-map signals within the HLA region (chromosome 6:29 607 078–33 267 103 (b37)) to a specific HLA gene allele or amino acid change, we re-imputed our discovery samples using IMPUTE2 v2.3.1 with a reference panel that enabled imputation of 424 classical HLA alleles and 1276 amino acid changes as described in Jia *et al.* [27]. We then repeated the association testing as described earlier.

### Mapping association signals to putative causal genes

We used functional annotation and colocalisation with expression quantitative trait loci (eQTL) signals to identify putative causal genes at each signal.

Annotation of the variants in each credible set was performed using SIFT [28], PolyPhen-2 and CADD, all implemented using the Ensemble GRCh37 Variant Effect Predictor (VEP) [29], alongside FATHMM [30]. Variants were annotated as deleterious if they were labelled deleterious by SIFT, probably damaging or possibly damaging by PolyPhen-2, damaging by FATHMM (specifying the “Inherited Disease” option of the “Coding Variants” method and using the “Unweighted” prediction algorithm), or had a CADD scaled score ≥20.

We queried the sentinel variants in GTEx V8 [31] and BLUEPRINT [32] (see supplementary table S3 for list of tissues). We tested for colocalisation of GWAS and eQTL signals using coloc [33]; H4 >80% was used to define a shared causal variant for eQTL and GWAS signals.

### Associations with other phenotypes

To investigate whether the signals of association with sputum production were driven by underlying respiratory phenotypes of the cases, a look-up for each signal was undertaken for 14 respiratory or respiratory-related traits from GWAS results: moderate-to-severe asthma (n_cases_=5135, n_controls_=25 675) [20], lung function (forced expiratory volume in 1 s (FEV_1_), forced vital capacity (FVC), FEV_1_/FVC and peak expiratory flow (PEF)) (n=400 102) [19], respiratory infection (n_cases_=19 459, n_controls_=101 438) [34], chronic cough (n_cases_=15 213, n_controls_=94 731), chronic bronchitis (n_cases_=977, n_controls_=108 967), idiopathic pulmonary fibrosis (IPF) (n_cases_=2668, n_controls_=8591) [35], smoking traits (smoking age of onset (n=124 590), smoking cessation (n_cases_=141 649, n_controls_=27 321), smoking cigarettes per day (n=120 744) and smoking initiation (n_cases_=170 772, n_controls_=212 859)) and asthma (n_cases_=23 948, n_controls_=118 538) [36]. Smoking trait results were from the UK Biobank component of Jiang *et al.* [37]; chronic cough and chronic bronchitis were defined for this study using UK Biobank data (supplementary material). Where the sentinel variant was not available in the look-up dataset, we utilised an alternative variant from the credible set with the highest posterior probability of being causal. A Bonferroni adjustment for 84 association tests was applied requiring a p<5.95×10^−4^ for association to be classified as statistically significant. Imputed HLA gene allele or amino acid changes were used for signals in the HLA region.

To investigate associations of the chronic sputum-associated variants with a wider range of phenotypes, we performed a PheWAS for 2172 traits in UK Biobank (false discovery rate <0.01; supplementary material) and searched the Open Targets Genetics Portal (p<5×10^−8^, version 0.4.0 (bd664ca); accessed 16 April 2021 [38]). PheWAS for imputed HLA alleles was performed using DeepPheWAS (supplementary material) [39].

### Sensitivity analyses

To further investigate whether the effects of the variants associated with risk of chronic sputum production differ between ever- and never-smokers, or between individuals with and without a history of chronic respiratory disease (spirometry-defined COPD GOLD 1–4, doctor-diagnosed asthma or doctor-diagnosed chronic bronchitis), we tested the association of sentinel variants in ever- and never-smokers and those with and without evidence of chronic respiratory disease separately. We additionally evaluated whether the associations differed between males and females or by the time of year of the survey (UK Biobank data-field 22500). Finally, we evaluated whether adjusting for current smoking (UK Biobank data-field 22506) (rather than ever- *versus* never-smoker status) affected the results.

## Results

**FIGURE 1:**
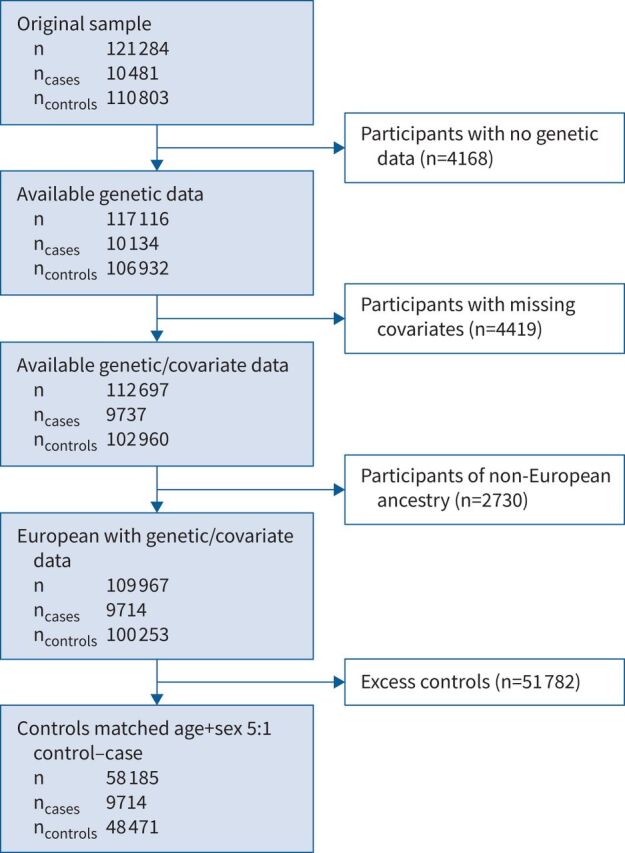
Study flowchart detailing case–control selection from the UK Biobank cohort.

**FIGURE 2:**
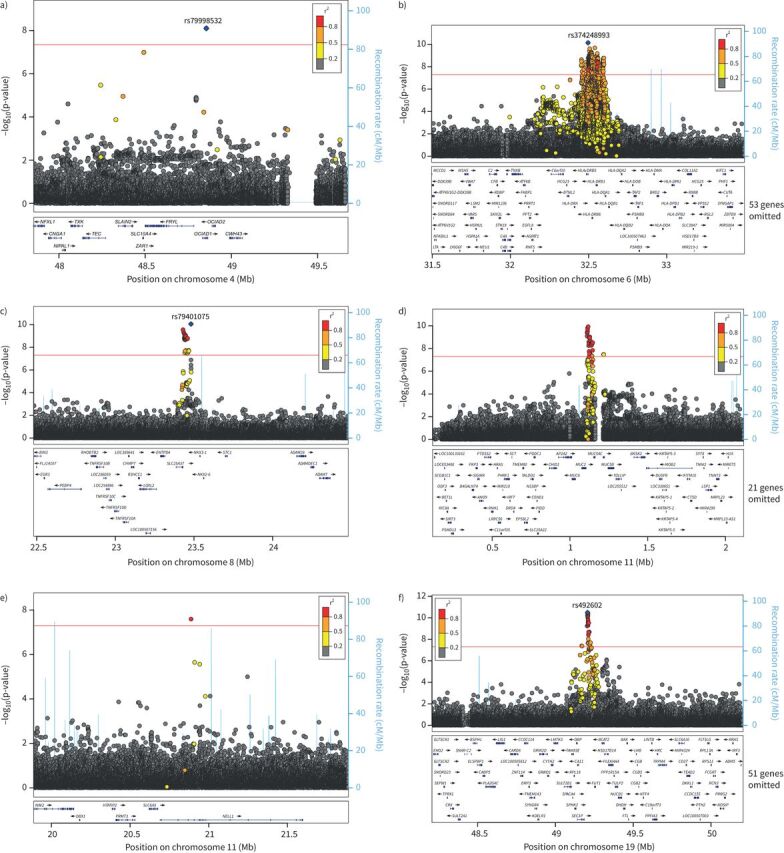
LocusZoom plots of the six sentinel signals: a) *OCIAD1* signal (rs79998532), b) *HLA-DRB1* signal (rs374248993), c) *NKX3-1* signal (rs79401075), d) *MUC2* signal (rs779167905), e) *NELL1* signal (rs529240826) and f) *FUT2* signal (rs492602).

A total of 10 481 participants answered “Yes” to the question “Do you bring up phlegm/sputum/mucus daily?” and 110 802 answered “No” (supplementary table S4). After excluding those with missing genotype and essential covariate data, and those of genetically determined European ancestry, a total of 9714 cases and 48 471 controls (figure 1) were included in the GWAS. Ever-smoking and respiratory disease were more common in the cases than in the controls (table 1). The genomic control inflation factor (λ) was 1.026, so no adjustments to the test statistics were applied (supplementary figure S1). Six independent novel signals met the genome-wide significance threshold of p<5×10^−8^ (table 2 and supplementary figure S2). These were four common variant signals (minor allele frequency >5%) in or near *MUC2*, *FUT2*, *HLA-DRB1* and *NKX3-1*, and two intronic rare variant signals (minor allele frequency <1%) in *OCIAD1* and *NELL1* (figure 2).

**TABLE 1:** Demographics and characteristics of cases and controls included in the genome-wide association study of chronic sputum production

	**Cases (n=9714)**	**Controls (n=48 471)**
**Mean age (years)**	57.7	57.7
**Female (%)**	42.5	42.5
**Smoking status**		
Ever-smoker	5306 (54.6)	20 912 (43.1)
Current smoker	983 (10.2)	1569 (3.2)
**Doctor-diagnosed chronic bronchitis**	407 (4.2)	416 (0.86)
**Doctor-diagnosed asthma**	2630 (27.1)	5251 (10.8)
**Cough on most days**	7022 (72.3)	3999 (8.3)
**Moderate-to-severe asthma**	520 (5.4)	521 (1.1)
**Self-reported chronic sinusitis**	181 (1.9)	1057 (2.2)
**Meets spirometry criteria for GOLD 1–4**	1511 (21.8)**^#^**	4766 (13.1)**^#^**

Data are presented as n (%), unless otherwise stated. COPD: Global Initiative for Chronic Obstructive Lung Disease. ^#^: n_cases_=6942 and n_controls_=36 321 with available spirometry that passed quality control.

**TABLE 2:** Novel genome-wide significant signals of association with chronic sputum production

**Chromosome:** **position (GRCh37)**	**rsID**	**Locus (distance from gene (bp))^#^**	**Coded/** **noncoded**	**Coded allele frequency (% (count))^¶^**		**OR (95 CI)**	**p-value**	**INFO^+^**	**Variants in 99% credible set (n (highest posterior probability))**
**4:48 854 355**	rs79998532	*OCIAD1* (intronic)	A/G	0.2 (233)	Discovery	2.36 (1.76–3.16)	8.00×10^−09^	0.92	3 (0.86)
					Replication	3.3 (0.11–98.6)	0.49		
					Meta-analysis	2.37 (1.77–3.17)	6.36×10^−09^		
**6:32 496 534**	rs374248993	*HLA-DRB1* ^§^	G/C	57 (66 355)	Discovery	1.12 (1.08–1.16)	7.30×10^−11^	0.87	*HLA*-DRB1*03:147^§^
					Replication	1.01 (0.84–1.21)	0.93		
					Meta-analysis	1.11 (1.08–1.15)	1.31×10^−10^		
**8:23 480 686**	rs79401075	*NKX3-1* (59 765)	A/G	10 (11 620)	Discovery	1.18 (1.12–1.24)	8.90×10^−11^	0.98	30 (0.32)
					Replication	1.20 (0.95–1.52)	0.12		
					Meta-analysis	1.18 (1.12–1.24)	2.65×10^−11^		
**11:1 116 931**	rs779167905	*MUC2* (12 513)	T/TTCTA	67 (78 158)	Discovery	1.12 (1.08–1.16)	1.20×10^−10^	0.98	30 (0.15)
					Replication	1.09 (0.93–1.28)	0.29		
					Meta-analysis	1.12 (1.08–1.15)	6.99×10^−11^		
**11:20 887 601**	rs529240826	*NELL1* (intronic)	GC/G	0.51 (588)	Discovery	1.91 (1.52–2.4)	2.50×10^−08^	0.67	2 (0.83)
					Replication	0.83 (0.38–1.78)	0.63		
					Meta-analysis	1.79 (1.44–2.22)	1.99×10^−07^		
**19:49 206 417**	rs492602	*FUT2* (exonic)	G/A	51 (58 803)	Discovery	1.11 (1.08–1.15)	3.20×10^−11^	1	32 (0.07)
					Replication	1.06 (1–1.14)	0.07		
					Meta-analysis	1.10 (1.07–1.13)	1.21×10^−11^		

^#^: start or end of nearest gene; ^¶^: values for discovery single nucleotide polymorphisms; ^+^: INFO score (imputation quality) taken from discovery; ^§^: amino acid change of threonine to arginine at codon 233 of exon 5 of *HLA-DRB1* (HLA gene allele HLA-DRB1*03:147).

No systematic differences were seen in effect sizes when stratifying by smoking status, by history of chronic respiratory disease, by sex, by time of year of survey or when including current smoking status as a covariate (supplementary table S5 and supplementary figures S3–S8) for the six sentinel variants. Through comparison of survey responses and linked primary care data we showed that primary care codes were not adequate proxies for the survey responses (supplementary material). We sought replication in five independent cohorts with a combined sample size of 1977 cases and 17 627 controls; data from all five replication cohorts were only available for the *FUT2* locus. Although none of the signals met criteria for significance in a meta-analysis of the replication cohorts, the directions of effect were consistent with the discovery results for the signals in or near *MUC2*, *FUT2*, *OCIAD1*, *HLA-DRB1* and *NKX3-1*, and all except the signals at *NELL1* and *HLA-DRB1* also increased in significance when the replication and discovery results were meta-analysed (table 2, supplementary table S13 and supplementary figure S9).

### Novel associations with chronic sputum production

#### HLA locus

The HLA signal was fine-mapped to an amino acid change of threonine to arginine (frequency 36.8%) at codon 233 of exon 5 of *HLA-DRB1* (HLA-DRB1*03:147) that was associated with decreased risk (OR 0.91 (95% CI 0.88–0.94)) of chronic sputum production (p=3.43×10^−9^). The amino acid change was in linkage disequilibrium with the GWAS sentinel variant rs374248993 (linkage disequilibrium r^2^=0.74 with HLA-DRB1*03:147) and the signal for rs374248993 was attenuated when conditioned on the amino acid change (supplementary figures S10 and S11).

**FIGURE 3:**
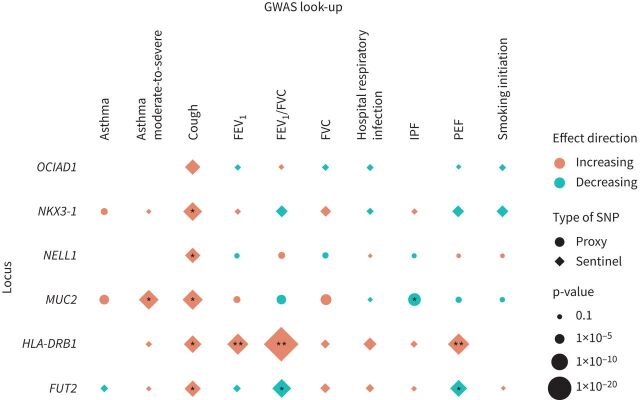
Results for association of sentinel variant risk alleles with respiratory traits. Results are aligned to the risk allele for chronic sputum production; effect direction “Increasing” can be read as increasing risk for binary traits and increasing values in quantitative traits. Chronic bronchitis and smoking age of onset, cigarettes per day and cessation phenotype look-ups were omitted as no associations with p<0.05 found. *: p<5.95×10^−4^ (Bonferroni adjustment for 84 association tests); **: p<5×10^−8^. Note that the idiopathic pulmonary fibrosis (IPF) association for rs779167905 (using proxy single nucleotide polymorphism (SNP) rs10902094) was attenuated when conditioned on rs35705950 (OR 0.99; p=0.784). FEV_1_: forced expiratory volume in 1 s; FVC: forced vital capacity; PEF: peak expiratory flow.

HLA-DRB1*03:147 was significantly associated with FEV_1_, FEV_1_/FVC and PEF at genome-wide significance (p<5×10^−8^) (figure 3 and supplementary table S6). The amino acid associated with increased risk of chronic sputum production (threonine) was associated with increased lung function; this had not been previously reported. The HLA PheWAS identified multiple significant associations for the HLA allele associated with increased risk of chronic sputum production with a wide range of quantitative traits (*e.g.* blood cell traits and liver biomarkers) and diseases (including decreased risk of gastrointestinal and thyroid-associated diseases, and increased risk of bronchiectasis and asthma) (supplementary table S7).

#### MUC2 locus

For the mucin locus signal (rs779167905 allele T), the allele associated with risk of chronic sputum production was also significantly associated with increased risk of asthma (OR 1.06; p=0.0027) and moderate-to-severe asthma (OR 1.13; p=6.3×10^−7^), increased FVC (β=0.0087; p=6×10^−4^) and decreased risk of IPF (OR 0.84; p=7.5×10^−6^) (figure 3 and supplementary table S6). There were no associations with gene expression for rs779167905 in GTEx or BLUEPRINT. However, we have previously shown that a proxy of rs779167905 (rs11602802, r^2^=0.94) was associated with mRNA levels of *MUC5AC* in bronchial epithelial brush samples collected from asthma patients, with the risk allele being associated with elevated *MUC5AC* expression [20].

Genome-wide significant associations with IPF [40] and moderate-to-severe asthma [20] have previously been reported at this chromosome 11 locus, so we undertook a conditional analysis to identify whether the chronic sputum production signal was independent of these previous signals. Repeating the association testing for this variant conditioning on the previously reported variants (rs35705950 [40] and rs11603634 [20]) identified that the chronic sputum production GWAS signal was independent of the IPF signal (rs779167905, conditional p=1.18×10^−10^) but was not independent of the previously reported moderate-to-severe asthma signal (rs779167905, conditional p=0.0039) (supplementary figures S12 and S13). Furthermore, the IPF association for rs779167905 (using proxy single nucleotide polymorphism (SNP) rs10902094) was also attenuated when conditioned on rs35705950 (OR 0.99; p=0.784).

Our PheWAS and Open Targets Genetics Portal analysis identified that the *MUC2* locus signal (rs779167905) allele that was associated with increased risk of chronic sputum production (allele T) was associated with higher risk of asthma and asthma-related traits in other studies [41–43] and with lower risk of gall bladder disease (supplementary tables S7 and S8).

#### FUT2 locus

The *FUT2* credible set included two variants that were annotated as functional using VEP. This included a stop-gain variant in *FUT2* (rs601338, linkage disequilibrium r^2^=0.992 with sentinel rs492602) and a nearby missense variant (rs602662, r^2^=0.882 with sentinel rs492602) that resulted in a glycine to serine amino acid change for the allele positively correlated with the chronic sputum production risk allele (supplementary tables S9 and S10).

**FIGURE 4:**
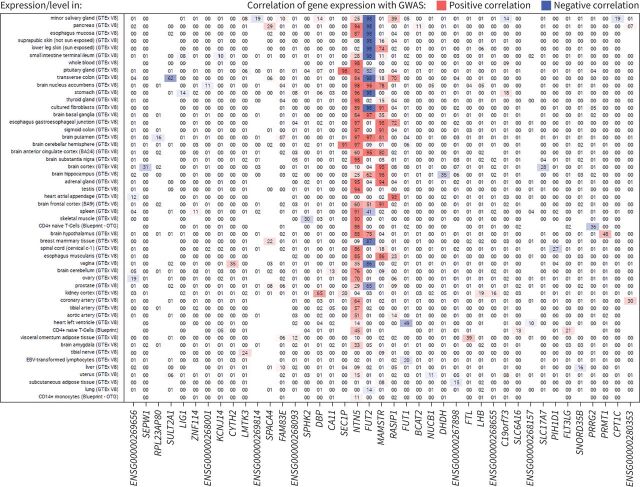
Results for expression quantitative trait loci colocalisation for the *FUT2* locus using variant rs492602. The numbers within the grid are the posterior probability of colocalisation (H4), with results aligned to the risk allele G for the rs492602 variant. Missing numbers indicate no data were available for the respective gene and tissue. GWAS: genome-wide association study; EBV: Epstein–Barr virus.

Sentinel variant rs492602 at the *FUT2* locus was associated with gene expression for *FUT2*, *NTN5*, *RASIP1*, *SEC1P* and *MAMSTR*, for which there was support for colocalisation of eQTL and GWAS signals in multiple tissues from GTEx V8 (figure 4 and supplementary table S11). Increased risk of chronic sputum production was consistently correlated with increased expression of *NTN5* and *MAMSTR* across a range of tissues. In contrast, the direction of the *FUT2* expression signal varied by tissue, with increased risk of chronic sputum production correlated with decreased expression of *FUT2* in brain tissues and with increased expression in gastrointestinal tissue. There were no associations in lung tissue and upper airway tissues were not available.

The sentinel variant for the *FUT2* region signal on chromosome 19 (rs492602) was associated with lung function measures FEV_1_/FVC and PEF (p=2.2×10^−6^ and p=1.1×10^−6^, respectively), with the chronic sputum production risk allele (G) associated with decreased lung function (figure 3 and supplementary table S6).

Our PheWAS and Open Targets Genetics Portal analysis for this variant identified 141 associations spanning multiple disease areas, phenotypes and biomarkers (supplementary tables S7 and S8). In summary, the allele associated with increased risk of chronic sputum production was associated with increased risk of gallstones [42, 44, 45], type 1 diabetes [46] and Crohn's disease [47–50], elevated vitamin B12 [51–54] and cholesterol and fat metabolites [41, 42, 55–59], hypertension/cardiovascular disease [41, 42, 44], excess alcohol with associated sequelae [44, 60–62], and increased risk of mumps and lower risk of childhood ear infections [63]. Higher risk of chronic sputum production was also associated with higher levels of γ-glutamyl transferase, total bilirubin and aspartate amino transferase, and lower levels of alanine aminotransferase and alkaline phosphatase.

#### Other novel loci

Using functional annotation of variants and eQTL analysis, no putative causal genes could be assigned to the signals in or near *OCIAD1* and *NELL1*. There was a single colocalising eQTL for *SLC25A37* in the *NKX3-1* locus with increased risk of chronic sputum production associated with a reduced expression of *SLC25A37* in brain cortex (supplementary table S11 and supplementary figure S14).

## Discussion

We describe a GWAS of chronic sputum production to identify genome-wide significant signals, and our novel findings implicate genes involved in mucin production and fucosylation, as well as the HLA class II histocompatibility antigen, HLA-DRB1. We provide functional evidence that the SNP signals we identify are associated with gene expression of *FUT2*, *MUC5AC* and *SLC5A37*.

Smoking is believed to be the main cause of excess sputum production, and is also associated with chronic infections, reduced lung function and susceptibility to chronic respiratory disease. Through identification of genetic association signals that are independent of smoking and history of chronic respiratory disease, our study demonstrates the value in studying a disease-relevant phenotype in a very large population that is agnostic to respiratory disease or smoking status.

The most significant signal implicated the gene *FUT2* which has been widely studied for its role in blood group antigen expression and association with gastric and respiratory infection. *FUT2* encodes fucosyltransferase 2 which mediates the transfer of fucose to the terminal galactose on glycan chains of cell surface glycoproteins and glycolipids. FUT2 creates a soluble precursor oligosaccharide called the H antigen, FuC-α((1,2)Galβ-), which is an essential substrate for the final step in the soluble ABO blood group antigen synthesis pathway. The *FUT2* locus allele associated with increased risk of chronic sputum production in this study is correlated with a nonsense allele that leads to inactivated FUT2 which results in a nonsecretory phenotype of ABO(H) blood group antigens [64] for homozygous carriers. This nonsense allele (rs601338 allele A) has frequencies of 25–50% in South Asian, European and African populations, but is rare (<1%) in East Asian populations [65]. Candidate gene studies of this locus have identified that nonsecretors (at increased risk of chronic sputum production according to our study) have a lower risk of *Helicobacter pylori* infection [66], rotavirus A infection [67, 68], norovirus infection [69–71], infant (12–24 months) respiratory illness [72], asthma exacerbations [73], otitis media [74], exacerbation in non-cystic fibrosis bronchiectasis and *Pseudomonas aeruginosa* airway infection in the same group [75], some evidence of slower HIV progression [71], and a higher risk of pneumococcal and meningococcal infection [76]. The T allele of another variant in high linkage disequilibrium at this locus (rs681343, r^2^=0.996 with rs492602), associated with increased risk of chronic sputum production in our study, was recently reported to be associated with increased risk of human polyomavirus 1 (BKV) virus infection, as measured by antibody response [77]. A recent GWAS of critically ill cases of coronavirus disease 2019 (COVID-19) (n_cases_=7491) showed that the risk allele for chronic mucus production (G) of rs492602 was protective against life-threating COVID-19 (OR 0.88 (95% CI 0.87–0.90); p=4.55×10^−9^) [78]. However, this finding was not replicated in the latest COVID-19 Host Genetics Initiative results for a similar phenotype [79]. The differing directions of effect of this signal on different phenotypes may be explained by the SNP effects on FUT2 expression which differ across cell and tissue types. Further targeted experiments in relevant cell and tissue types would be needed to elucidate this and define the likely effects of targeting FUT2 directly or indirectly.

Epitopes that are fucosylated by FUT2 play a role in cell–cell interaction, including host–microbe interaction [80, 81], and mediate interaction with intestinal microbiota, thereby influencing its composition [82–85]. While there has been no direct evidence of host–pathogen binding on the FUT2 generated epitopes for nongastrointestinal infection, there is evidence that FUT2 can influence nonbinding ligands such as sialic acid [86]. Sialic acid binding has been shown to be important for adenovirus binding in cell models [87] and modulating this binding has been implicated as a possible mechanism for increasing risk of mumps infection [63].

FUT2 may also be key to the function of mucins, including those encoded by genes at our other significant locus (*i.e. MUC2*, *MUC5AC* and *MUC5B*). Mucins are a major constituent of airway mucus and MUC5AC is major gel-forming mucin secreted by airway epithelial cells. FUT2 may play a key role in MUC5AC regulation leading to excess mucus production or its increased viscosity, a common characteristic observed in patients with airway obstructive diseases including asthma, bronchitis and COPD. Analysis of oligosaccharides released from insoluble colonic mucins, largely Muc2, by mass spectrometry shows complete lack of terminal fucosylation of *O-*linked oligosaccharides in Fut2-LacZ-null mice [88]. FUT2 has also been shown to determine the *O-*glycosylation pattern of Muc5ac in mice [89]. The significant signal at *MUC2* in our analysis was not independent of the previously reported moderate-to-severe asthma signal [20] for which *MUC5AC* was implicated as the most likely causal gene using gene expression data from bronchial epithelial cells. In that study we went on to show that the signal (rs11602802 used as proxy) was associated with mRNA levels of MUC5AC in bronchial epithelial brush samples collected from asthma patients, with the risk allele being associated with elevated MUC5AC. There was also a nonsignificant trend for MUC5B to have a reduced mRNA level in the presence of the moderate-to-severe asthma risk allele. These *ex vivo* observations have recently been replicated in nasal epithelial cell brush samples in an independent cohort and extended to show this signal (rs12788104 within the credible set of *MUC2* signal) regulates MUC5AC protein levels *in vitro* using nasal epithelial cells from genotyped subjects in the air–liquid interface model [90]. Although our analysis did not identify an association at the *MUC2* locus with COPD-related traits (FEV_1_ and FEV_1_/FVC), a recent study has also highlighted MUC5AC as a potential biomarker for COPD prognosis [91].

The particular allele that was found to explain the association signal in the HLA region (HLA-DRB1*03:147 [92]) has only recently been reported and so there is limited information about functionality. Associations of this allele with other GWAS traits should be interpreted with caution given the high linkage disequilibrium across the region. Furthermore, the association of this allele with increased sputum production and increased lung function reminds us that increased sputum production is part of the adaptive immune response to environmental insult and approaches to target mucus production must also consider potential negative effects of reducing sputum production.

We only report overlap of chronic sputum production association signals with association signals for gene expression regulation where there is statistical support that these signals share a causal variant. In addition to a comprehensive PheWAS, we provide a deeper assessment of associations with relevant respiratory phenotypes that highlights previously unreported associations with lung function for the *HLA-DRB1* and *FUT2* signals.

As only a subset of UK Biobank participants provided answers to the sputum production question, we expected that we might be able to define a replication case–control dataset from the remaining >300 000 participants using primary care data. However, evaluation of the positive predictive value of primary care codes for sputum production, when compared with the questionnaire data, was very low (supplementary material). This could reflect a low utilisation of sputum codes in primary care or that participants have not reported this symptom to their general practitioner. We obtained supportive evidence for four of the signals utilising data from five general population cohorts. The limited sample size (the case sample size for replication was 23% of the size available for discovery) impacted our ability to show statistically significant replication. Furthermore, we note that, for three of the replication cohorts (LifeLines 1, LifeLines 2 and Vlagtwedde-Vlaardingen), the sputum production question asked specifically about winter symptoms, while the UK Biobank survey did not restrict to any specific season. However, given the strong evidence summarised earlier for the involvement of the probable causal genes in control of pathways relevant to mucus production, we believe the associations identified are highly likely to be real. Due to very low numbers, we were unable to evaluate the effects of these signals in individuals of non-European ancestry, thereby limiting the generalisability of our findings to non-European ancestry groups. Efforts are urgently needed to improve diversity in genomics research [93] such as the planned Our Future Health initiative in the UK. In summary, the HLA, *MUC2* and *FUT2* loci show strong candidacy for a role in sputum production, with overlap with infection and related phenotypes and known mechanistic interactions between the genes at the *FUT2* and *MUC2* loci, suggesting that these signals are likely to be robust. The large number of associations of the *FUT2* locus with a broad array of phenotypes, tissue-dependent expression of *FUT2* and association with expression of other genes in the region may have implications for drug targeting guided by this locus. Experimental studies to characterise the specific interplay between FUT2 activity and mucin genes expressed in the airways are warranted.

### Conclusions

Chronic sputum production is a phenotype characteristic of several respiratory diseases, as well as being a common cause for referrals in the absence of overt disease, and is of interest for pharmaceutical intervention. We report novel genetic factors which influence chronic sputum production and these signals highlight fucosylation of mucin as a driving factor of chronic sputum production. These signals could provide insight into the molecular pathways of sputum production and represent potential future targets for drug development [94].

## Supplementary material

10.1183/13993003.01667-2022.Supp1 **Please note:** supplementary material is not edited by the Editorial Office, and is uploaded as it has been supplied by the author.
Supplementary text, figures and table captions ERJ-01667-2022.Supplement
STREGA reporting recommendations ERJ-01667-2022.STREGA
Supplementary tables ERJ-01667-2022.Tables

## Shareable PDF

10.1183/13993003.01667-2022.Shareable1 This one-page PDF can be shared freely online.
Shareable PDF ERJ-01667-2022.Shareable
