# Supplementary material for: Genome-wide association study of chronic sputum production implicates loci involved in mucus production and infection
Source: Eur Respir J. 2023 Jun 15;61(6):2201667. doi: 10.1183/13993003.01667-2022 (PMC10284065; doi:10.1183/13993003.01667-2022)
Supplement: Supplementary file 4 [file ERJ-01667-2022.Shareable.pdf]

EUROPEAN RESPIRATORY JOURNAL
ORIGINAL RESEARCH ARTICLE
R.J. PACKER ET AL.

SHAREABLE PDF

Genome-wide association study of chronic sputum production implicates loci involved in mucus production and infection

Richard J. Packer [1,2], Nick Shrine [1], Robert Hall[3], Carl A. Melbourne[1], Rebecca Thompson[3], Alex T. Williams[1], Megan L. Paynton[1], Anna L. Guyatt [1], Richard J. Allen[1], Paul H. Lee [1], Catherine John[1,2], Archie Campbell[4], Caroline Hayward[5], Maaike de Vries [6], Judith M. Vonk [6], Jonathan Davitte [7], Edith Hessel [8], David Michalovich [8], Joanna C. Betts[8], Ian Sayers[3], Astrid Yeo[8], Ian P. Hall[3], Martin D. Tobin [1,2] and Louise V. Wain [1,2]

[1]Department of Population Health Sciences, University of Leicester, Leicester, UK. [2]Leicester NIHR Biomedical Research Centre, Glenfield Hospital, Leicester, UK. [3]Centre for Respiratory Research, NIHR Nottingham Biomedical Research Centre, School of Medicine, Biodiscovery Institute, University of Nottingham, Nottingham, UK. [4]Centre for Genomic and Experimental Medicine, Institute of Genetics and Cancer, University of Edinburgh, Western General Hospital, Edinburgh, UK. [5]Medical Research Council Human Genetics Unit, Institute of Genetics and Cancer, University of Edinburgh, Edinburgh, UK. [6]University of Groningen, University Medical Center Groningen, Department of Epidemiology and Groningen Research Institute for Asthma and COPD (GRIAC), Groningen, The Netherlands. [7]GSK R&D, Collegeville, PA, USA. [8]GSK R&D, Stevenage, UK.

Corresponding author: Richard J. Packer (richard.packer@leicester.ac.uk)

Check for updates

Shareable abstract (@ERSpublications)
Genome-wide association study in UK Biobank identifies six novel loci associated with chronic sputum production at genome-wide significance in a disease agnostic population. These include a *FUT2* locus, highlighting a possible target for drug development. https://bit.ly/3IRVJeT

Cite this article as: Packer RJ, Shrine N, Hall R, *et al*. Genome-wide association study of chronic sputum production implicates loci involved in mucus production and infection. *Eur Respir J* 2023; 61: 2201667 [DOI: 10.1183/13993003.01667-2022].

This single-page version can be shared freely online.

Copyright ©The authors 2023.

This version is distributed under the terms of the Creative Commons Attribution Licence 4.0.

Received: 11 Jan 2022
Accepted: 17 Feb 2023

Abstract

Background Chronic sputum production impacts on quality of life and is a feature of many respiratory diseases. Identification of the genetic variants associated with chronic sputum production in a disease agnostic sample could improve understanding of its causes and identify new molecular targets for treatment.

Methods We conducted a genome-wide association study (GWAS) of chronic sputum production in UK Biobank. Signals meeting genome-wide significance ($p<5\times10^{-8}$) were investigated in additional independent studies, were fine-mapped and putative causal genes identified by gene expression analysis. GWASs of respiratory traits were interrogated to identify whether the signals were driven by existing respiratory disease among the cases and variants were further investigated for wider pleiotropic effects using phenome-wide association studies (PheWASs).

Results From a GWAS of 9714 cases and 48 471 controls, we identified six novel genome-wide significant signals for chronic sputum production including signals in the human leukocyte antigen (HLA) locus, chromosome 11 mucin locus (containing *MUC2*, *MUC5AC* and *MUC5B*) and *FUT2* locus. The four common variant associations were supported by independent studies with a combined sample size of up to 2203 cases and 17 627 controls. The mucin locus signal had previously been reported for association with moderate-to-severe asthma. The HLA signal was fine-mapped to an amino acid change of threonine to arginine (frequency 36.8%) in HLA-DRB1 (*HLA-DRB1*03:147*). The signal near *FUT2* was associated with expression of several genes including *FUT2*, for which the direction of effect was tissue dependent. Our PheWAS identified a wide range of associations including blood cell traits, liver biomarkers, infections, gastrointestinal and thyroid-associated diseases, and respiratory disease.

Conclusions Novel signals at the *FUT2* and mucin loci suggest that mucin fucosylation may be a driver of chronic sputum production even in the absence of diagnosed respiratory disease and provide genetic support for this pathway as a target for therapeutic intervention.
